# Supplementary material for: Association between Intraoperative Blood Pressure Drop and Clinically Significant Hypoperfusion in Abdominal Surgery: A Cohort Study
Source: J Clin Med. 2021 Oct 28;10(21):5010. doi: 10.3390/jcm10215010 (PMC8584611; doi:10.3390/jcm10215010)
Supplement: Supplementary file 1 [file jcm-10-05010-s001.zip › jcm-1408846-supplementary.pdf]

Table S1. STROBE Statement—Checklist of items that should be included in reports of ***cohort studies.***

	Item No	Recommendation
**Title and abstract**	1	(*a*) Indicate the study's design with a commonly used term in the title or the abstract page 1,2
		(*b*) Provide in the abstract an informative and balanced summary of what was done and what was found page 2
		**Introduction**
Background/rationale	2	Explain the scientific background and rationale for the investigation being reported page 4
Objectives	3	State specific objectives, including any prespecified hypotheses page 4
		**Methods**
Study design	4	Present key elements of study design early in the paper page 5-7
Setting	5	Describe the setting, locations, and relevant dates, including periods of recruitment, exposure, follow-up, and data collection page 5-7
Participants	6	(*a*) Give the eligibility criteria, and the sources and methods of selection of participants. Describe methods of follow-up page 5
		(*b*) For matched studies, give matching criteria and number of exposed and unexposed N/A
Variables	7	Clearly define all outcomes, exposures, predictors, potential confounders, and effect modifiers. Give diagnostic criteria, if applicable page 5-7
Data sources/ measurement	8*	For each variable of interest, give sources of data and details of methods of assessment (measurement). Describe comparability of assessment methods if there is more than one group page 6-7
Bias	9	Describe any efforts to address potential sources of bias page 5-7
Study size	10	Explain how the study size was arrived at page 5
Quantitative variables	11	Explain how quantitative variables were handled in the analyses. If applicable, describe which groupings were chosen and why page 7
Statistical methods	12	(*a*) Describe all statistical methods, including those used to control for confounding page 7
		(*b*) Describe any methods used to examine subgroups and interactions page 7
		(*c*) Explain how missing data were addressed N/A
		(*d*) If applicable, explain how loss to follow-up was addressed N/A
		(*e*) Describe any sensitivity analyses N/A
		**Results**
Participants	13*	(a) Report numbers of individuals at each stage of study—eg numbers potentially eligible, examined for eligibility, confirmed eligible, included in the study, completing follow-up, and analysed Figure 1
		(b) Give reasons for non-participation at each stage Figure 1
		(c) Consider use of a flow diagram Figure 1
Descriptive data	14*	(a) Give characteristics of study participants (eg demographic, clinical, social) and information on exposures and potential confounders page 8, Table 1, Table 2
		(b) Indicate number of participants with missing data for each variable of interest N/A
		(c) Summarise follow-up time (eg, average and total amount) N/A
Outcome data	15*	Report numbers of outcome events or summary measures over time page 7
Main results	16	(*a*) Give unadjusted estimates and, if applicable, confounder-adjusted estimates and their precision (eg, 95% confidence interval). Make clear which confounders were

		adjusted for and why they were included page 8-12, Table 1,2,3,4,5, Supplementary Material
		(*b*) Report category boundaries when continuous variables were categorized page 8-12
		(*c*) If relevant, consider translating estimates of relative risk into absolute risk for a meaningful time period N/A
Other analyses	17	Report other analyses done—eg analyses of subgroups and interactions, and sensitivity analyses N/A
	**Discussion**	
Key results	18	Summarise key results with reference to study objectives page 13
Limitations	19	Discuss limitations of the study, taking into account sources of potential bias or imprecision. Discuss both direction and magnitude of any potential bias page 13-16
Interpretation	20	Give a cautious overall interpretation of results considering objectives, limitations, multiplicity of analyses, results from similar studies, and other relevant evidence page 13-16
Generalisability	21	Discuss the generalisability (external validity) of the study results page 15
	**Other information**	
Funding	22	Give the source of funding and the role of the funders for the present study and, if applicable, for the original study on which the present article is based 17

* Give information separately for exposed and unexposed groups.

Table S2. Types of surgery.

**Type of surgery**	
Surgery of the pancreas	102 (20%)
Surgery of the small intestine	74 (14.6%)
Surgery of the large intestine	97 (19.1%)
Cholecystectomy	113 (22.2%)
Hernia repair surgery	56 (11%)
Gastric surgery	25 (5%)
Surgery of the esophagus	8 (1.6%)
Liver surgery	8 (1.6%)
Splenectomy	3 (0.6%)
Other abdominal surgery	22 (4.3%)

Table S3. Multivariate logistic regression models in predicting the occurrence of hypoperfusive outcome.

**Variable*** ↓ **Model** →	> 0 episodes of MAP< 55 mmHg	> 0 episodes of MAP< 60 mmHg	> 1 episodes of MAP< 65 mmHg	> 4 episodes of MAP< 70 mmHg	> 17 episodes of MAP< 75 mmHg	> 27 episodes of MAP drop > 20% from baseline	> 20 episodes of MAP drop > 25% from baseline	> 2 episodes of MAP drop > 30% from baseline	> 4 episodes of MAP drop > 35% from baseline	> 0 episodes of MAP drop > 40% from baseline
IOH threshold	2.56 (1.05-6.26)	2.61 (1.22-5.59)	2.50 (1.17-5.30)	2.67 (1.26-5.67)	*	*	*	*	*	*
Chronic arterial hypertension (1/0)	3.20 (1.05-7.23)	3.57 (1.56-8.20)	3.77 (1.63-8.69)	3.81(1.65-8.81)	2.98 (1.33-6.68)	2.98 (1.33-6.68)	2.98 (1.33-6.68)	2.98 (1.33-6.68)	2.98 (1.33-6.68)	2.98 (1.33-6.68)
Procedure duration (per 1 minute)	1.006 (1.003-1.008)	1.006 (1.004-1.009)	1.006 (1.003-1.008)	1.006 (1.003-1.008)	1.006 (1.004-1.009)	1.006 (1.004-1.009)	1.006 (1.004-1.009)	1.006 (1.004-1.009)	1.006 (1.004-1.009)	1.006 (1.004-1.009)
Chronic Kidney Disease (1/0)	5.13 (1.33-19.80)	5.16 (1.32-20.16)	4.76 (1.19-19.09)	4.7 (1.22-18.22)	5.60 (1.49-21.14)	5.60 (1.49-21.14)	5.60 (1.49-21.14)	5.60 (1.49-21.14)	5.60 (1.49-21.14)	5.60 (1.49-21.14)
AUROC; p-value	0.836 (0.801-0.867); p<0.000	0.831 (0.795-0.863); p<0.0001	0.824 (0.788-0.856); p<0.0001	0.833 (0.797-0.864; p<0.0001	0.816 (0.779-0.849); p<0.0001	0.816 (0.779-0.849); p<0.0001	0.816 (0.779-0.849); p<0.0001	0.816 (0.779-0.849); p<0.0001	0.816 (0.779-0.849); p<0.0001	0.816 (0.779-0.849); p<0.0001

Values are presented as odds ratios (confidence intervals). "*" IOH thresholds that failed to be included in the multivariable models.
